# Catastrophic outcome following misidentification of bowel anatomy during Hartmann's reversal: A case report and technical considerations

**DOI:** 10.1016/j.ijscr.2024.110633

**Published:** 2024-11-19

**Authors:** Asim M. Almughamsi

**Affiliations:** Department of Surgery, Taibah University, Medina, Saudi Arabia

**Keywords:** Hartmann procedure, Surgical anastomosis, Postoperative complications, Intestinal fistula, Adhesions, Case report

## Abstract

**Background:**

Reversal of Hartmann's procedure is a complex surgery with potential complications. This case report describes a rare and severe complication following an attempted reversal.

**Case presentation:**

A 53-year-old male who had undergone a Hartmann's procedure for non-metastatic sigmoid colon cancer presented with bowel obstruction 10 days after attempted reversal surgery at another hospital. Imaging studies suggested an entero-colic fistula. Emergency laparotomy revealed dense adhesions and multiple bowel injuries. The procedure was terminated, and controlled fistulae were created.

**Management and outcome:**

The patient required two months of intensive care. A subsequent surgery excised the fistulae and restored intestinal continuity, leaving the patient with an end colostomy and approximately 120 cm of ileum.

**Conclusion:**

This case highlights the potential risks of Hartmann's reversal and emphasizes the importance of proper patient selection, timing, and surgical expertise. It underscores the need for thorough preoperative evaluation and preparation when attempting such complex surgeries.

## Highlights

• Complex Hartmann's reversal led to entero-colic fistula requiring staged reconstruction.
• Misidentification of bowel anatomy during reversal caused severe complications.
• The patient recovered after a two-month ICU stay and subsequent reconstructive surgery.
• The case demonstrates the critical need for expert colorectal surgeons in Hartmann's reversals.

## 1 Introduction

Hartmann's procedure (HP) is a life-saving surgical intervention commonly performed in emergencies involving the sigmoid colon. While initially designed as a two-stage procedure, HP reversal to restore intestinal continuity is often challenging and associated with significant morbidity. Recent literature reports reversal rates ranging from 23 % to 50 %, with higher rates observed in cases of benign disease. The complexity of HP reversal stems from multiple factors, including dense adhesions, altered anatomy, and potential complications from the initial surgery or underlying condition. These challenges necessitate careful patient selection, meticulous preoperative planning, and execution by experienced surgeons, preferably those with colorectal subspecialty training. This case report presents a rare and severe complication following an attempted HP reversal, highlighting the potential risks and technical difficulties associated with this procedure. We describe a patient who developed catastrophic complications due to misidentification of bowel anatomy during reversal surgery, resulting in multiple enterotomies, fistula formation, and prolonged intensive care. By sharing this experience, we emphasize the importance of proper surgical technique, the need for thorough preoperative evaluation, and the critical role of surgeon experience in managing these complex cases. This report also reminds the surgical community of the potential pitfalls in HP reversal and strategies to mitigate risks. This work has been reported in line with the SCARE 2023 criteria [1].

## 2 Case presentation

### 2.1 Patient information

A 53-year-old male presented to the Emergency Department with a 10-day history of abdominal colic, distension, vomiting, and absolute constipation. The patient had undergone a Hartmann's procedure for non-metastatic sigmoid colon cancer 8 months prior, followed by 6 months of adjuvant chemotherapy. One week before the presentation, he had undergone HP reversal at another institution.

### 2.2 Patient history and presentation

The initial Hartmann's procedure was performed as an emergency surgery due to obstructing sigmoid cancer with localized perforation. The procedure was completed successfully with no immediate post-operative complications. The patient completed six cycles of FOLFOX chemotherapy with good tolerance. The pre-reversal assessment included a CT scan showing no evidence of recurrence, colonoscopy confirming a healthy rectal stump, and nutritional optimization with serum albumin of 3.8 g/dL. The patient's BMI was 24.2 kg/m^2^, and he had no significant comorbidities that would contraindicate reversal surgery.

### 2.3 Clinical findings

On examination, the patient was conscious, alert, and oriented but in evident discomfort. Vital signs were stable. Abdominal examination revealed distention with central and lower abdominal tenderness.

### 2.4 Timeline

- 8 months before presentation: Hartmann's procedure for non-metastatic sigmoid colon cancer
- 2 months before presentation: Completion of adjuvant chemotherapy
- 10 days before the presentation: Attempted Hartmann's reversal at another institution
- Day 0: Presentation to Emergency Department with symptoms of bowel obstruction.

### 2.5 Diagnostic assessment

Laboratory investigations were unremarkable except for leukocytosis (14,400/mm^3^) and elevated ESR (38 mm/h).

**Fig. 1:**
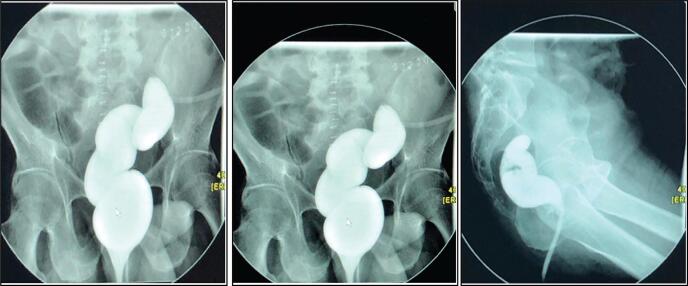
Gastrografin enema demonstrating closed rectal stump and abnormal contrast progression.

Abdominal radiography demonstrated dilated small bowel loops with multiple air-fluid levels. A contrast-enhanced CT scan revealed contrast progression from the stomach to jejunal loops, then directly into the left colon without opacification of the right colon. The ileal loops and right colon appeared distended and contrast-filled, while the descending colon collapsed to the level of the anastomosis. These findings suggested an entero-colic fistula. Intra-abdominal fat stranding and minimal free fluid were also noted. A subsequent Gastrografin enema confirmed a closed rectal stump (Fig. 1).

### 2.6 Therapeutic Intervention

**Fig. 2:**
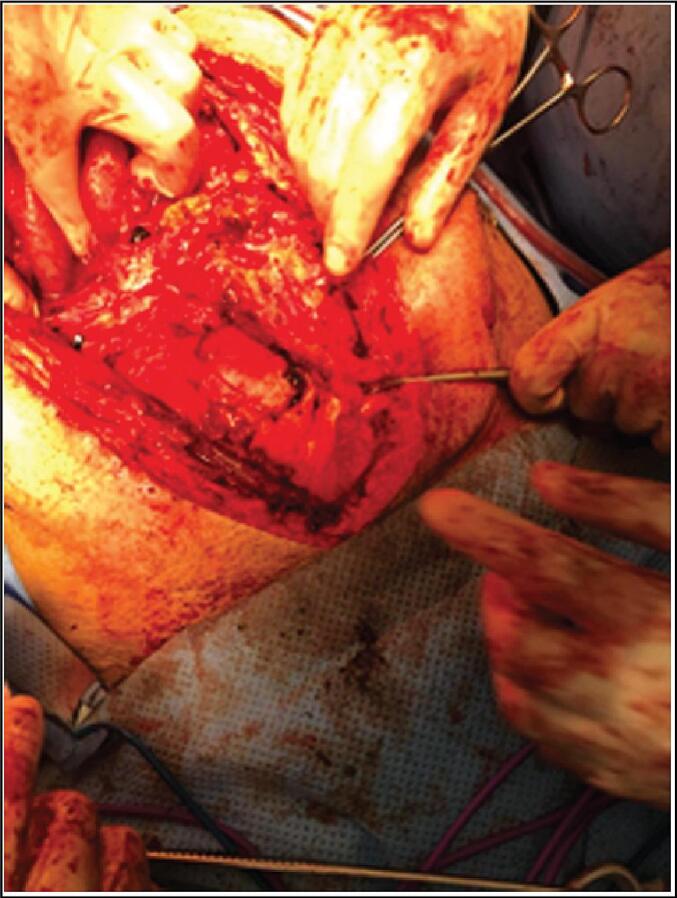
Intraoperative photograph showing dense adhesions encountered during emergency laparotomy.

**Fig. 3:**
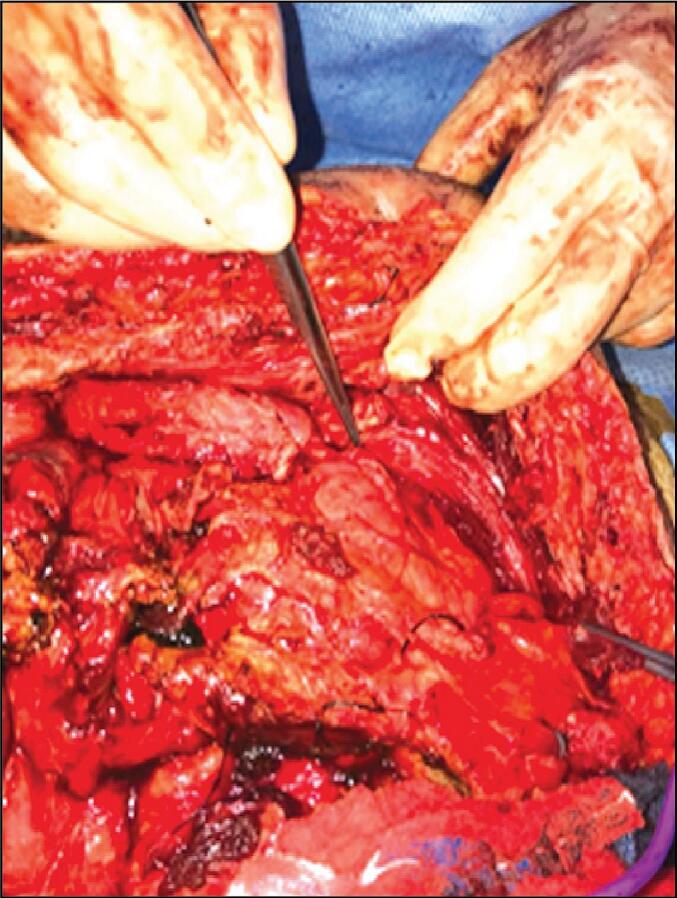
Multiple enterotomies identified during adhesiolysis.

Given the clinical and radiological findings indicative of intestinal obstruction, the patient was prepared for emergency exploratory laparotomy. Upon entering the abdomen, dense adhesions were encountered, making exploration extremely difficult (Fig. 2). Attempts at dissection resulted in multiple enterotomies due to the friability of the bowel loops (Fig. 3).

**Fig. 4:**
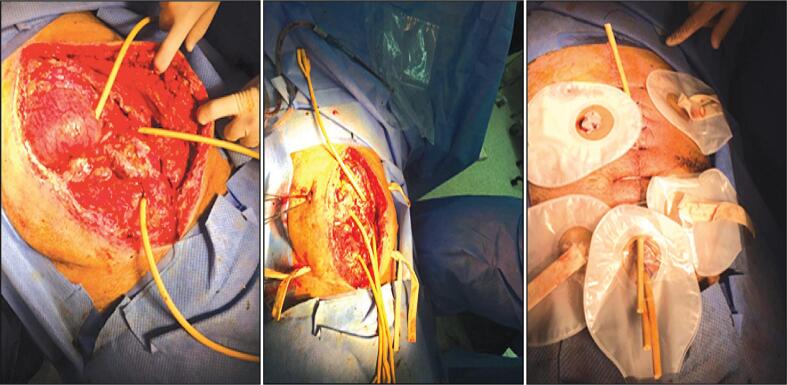
Creation of controlled fistulae using Foley catheters and right-sided decompressing colostomy.

The procedure was terminated, and controlled fistulae were created using Foley catheters. A right-sided decompressing colostomy with a mucous fistula was also constructed (Fig. 4).

Sharp and blunt dissection techniques were employed for adhesiolysis, with particular attention to maintaining a close dissection plane along the bowel serosa to minimize inadvertent enterotomies. Despite these precautions, the extreme friability of the tissues led to multiple small bowel injuries. The controlled fistulae were created using 24Fr Foley catheters secured with purse-string sutures of 3–0 polypropylene, with the balloon inflated to 10 mL. The catheters were brought out through separate skin incisions and connected to drainage bags for output measurement.

### 2.7 Follow-up and outcomes

The patient required two months of intensive care following the emergency surgery. Subsequent surgery was performed to excise the fistulae and restore intestinal continuity. This procedure left the patient with an end colostomy and approximately 120 cm of ileum.

Nutritional management was particularly challenging during the prolonged ICU stay. The patient required total parenteral nutrition (TPN) for 6 weeks, carefully titrated to maintain positive nitrogen balance while monitoring liver function. Following the reconstructive surgery, a staged approach to enteral feeding was implemented, starting with elemental formula via jejunostomy tube before transitioning to oral intake. At 6-month follow-up, the patient had regained 85 % of his baseline weight and reported satisfactory quality of life despite the permanent colostomy, with a Gastrointestinal Quality of Life Index (GIQLI) score of 105. He has successfully returned to modified work duties and manages his colostomy independently.

**Fig. 5:**
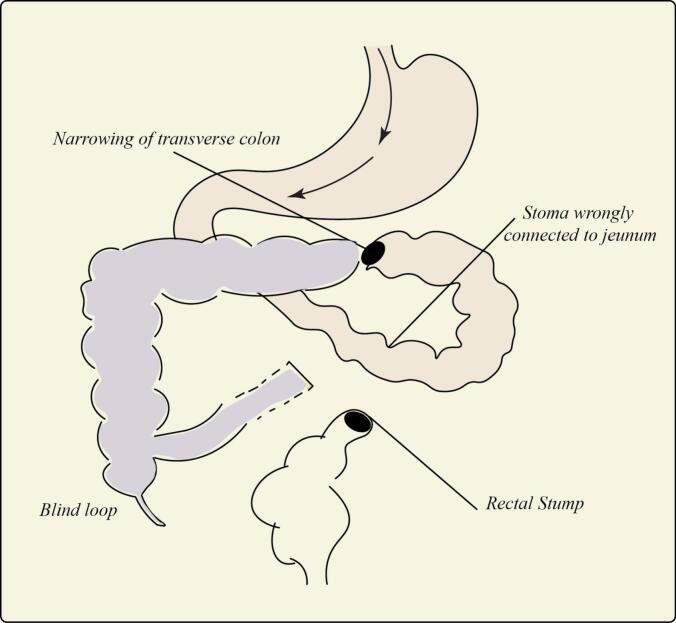
Schematic diagram illustrating the disturbed anatomy following the initial Hartmann's reversal attempt.

Post-operatively, a diagram was constructed to illustrate the suspected disturbed anatomy following the HP reversal, which had resulted in intestinal obstruction (Fig. 5).

### 2.8 Patient perspective

The patient expressed profound distress over the complications and prolonged hospital stay. He stated, “I never imagined a surgery to improve my quality of life could lead to such severe complications. The experience has been physically and emotionally challenging, but I'm grateful for the care I've received and hopeful for continued improvement.”

## 3 Discussion

This case report illustrates a severe complication following an attempted reversal of Hartmann's procedure (HP), highlighting the complex nature of this surgery and the potential for catastrophic outcomes when anatomical landmarks are misidentified.

Reversal of HP is known to be a challenging procedure, with reported reversal rates ranging from 23 % to 50 % in various studies [[2], [3], [4]]. The lower reversal rate is attributed to multiple factors, including patient comorbidities, technical difficulties, and the high risk of complications [5]. In our case, the reversal was attempted 8 months after the initial surgery, which is generally considered an acceptable timeframe [6].

The primary complication in this case appears to stem from a misidentification of bowel anatomy during the reversal procedure. The operating surgeon likely encountered a frozen abdomen with dense adhesions, a common finding in post-HP patients [7]. In an attempt to restore bowel continuity, it seems the surgeon inadvertently anastomosed the end sigmoid colostomy to the proximal ileum instead of the rectal stump. This error led to a complete bypassing of a significant portion of the small intestine and the entire right colon, resulting in malabsorption and obstruction.

The extensive adhesions encountered during the emergency laparotomy are typical in patients undergoing HP reversal [8]. However, the extreme friability of the bowel observed in our patient, leading to multiple enterotomies, suggests a particularly severe inflammatory response. This underscores the importance of meticulous dissection techniques and the potential need for extensive adhesiolysis in such cases [9].

The decision to create controlled fistulae and a decompressing colostomy in our patient was a life-saving measure in the face of non-reconstructable bowel injury. This approach aligns with damage control principles in acute care surgery, prioritizing patient survival over immediate anatomical reconstruction [10].

The prolonged ICU stay and the need for multiple surgeries, in this case, highlight the potential for significant morbidity following complications of HP reversal. This aligns with the literature that reports that overall postoperative complication rates range from 16.1 % to 41 % following HP reversal [11,12]. Similar cases of anatomical misidentification during Hartmann's reversal have been reported, though rarely with such severe consequences [13,14].

This case emphasizes several crucial points for surgeons contemplating HP reversal:

1. Preoperative Planning: Thorough preoperative imaging, including CT scans and contrast studies, is essential to delineate the altered anatomy and plan the surgical approach [15].
2. Surgical Expertise: HP reversal should ideally be performed by experienced colorectal surgeons familiar with the procedure's potential pitfalls [16].
3. Intraoperative Strategies: In cases of dense adhesions, techniques such as fluoroscopy-guided rectal stump identification or lighted ureteral stents can aid in correct anatomical identification [17].
4. Patient Selection: Careful patient selection, considering factors such as nutritional status, extent of adhesions, and comorbidities, is crucial in minimizing postoperative complications [18].
5. Informed Consent: Patients should be thoroughly counseled about the high risk of complications associated with HP reversal [19].

## 4 Conclusion

This case report highlights a rare but severe complication following the reversal of a Hartmann's procedure, underscoring the complex nature of this surgery and the potential for catastrophic outcomes when anatomical landmarks are misidentified. It serves as a crucial reminder to the surgical community about the inherent risks of Hartmann's reversal and the importance of meticulous surgical technique. Several key lessons can be drawn from this experience: Hartmann's reversal should be considered a major surgical procedure requiring thorough preoperative evaluation and planning; it should ideally be performed by experienced surgeons, preferably those with colorectal subspecialty training; preoperative imaging studies are essential to delineate the altered anatomy; intraoperative challenges should be anticipated and managed carefully; surgeons should maintain a low threshold for modifying or aborting the procedure when faced with uncertain anatomy or extensive adhesions; and patients should be thoroughly counseled about the associated risks. This case also emphasizes the importance of a multidisciplinary approach in managing complications. In conclusion, while Hartmann's reversal remains an essential procedure for restoring intestinal continuity, this case serves as a sobering reminder of its potential risks, calling for continued education, careful patient selection, and ongoing efforts to improve surgical techniques and perioperative management strategies to enhance the safety and success of this challenging procedure.

## Patient consent

The patient provided written informed consent for the publication of this case report and accompanying images. The editor-in-chief of this journal can review a copy of the written permission upon request.

## Ethical approval

Ethical approval was not required for this case report as it does not contain any studies performed by the author with human participants or animals.

## Funding

This case report did not receive any specific grant from funding agencies in the public, commercial, or not-for-profit sectors.

## Guarantor

I, Dr. Asim M Almughamsi, accept full responsibility for this work and/or the conduct of the study, had access to the data, and controlled the decision to publish.

## Research registration number

1. Name of the registry: Not applicable
2. Unique identifying number or registration ID: Not applicable
3. Hyperlink to your specific registration: Not applicable.

## CRediT authorship contribution statement

Asim M Almughamsi was responsible for all aspects of this case report, including patient care, data collection, analysis, manuscript preparation, and review.

## Declaration of competing interest

The author declares no conflicts of interest in relation to this case report.
